# The effects of treatment with liraglutide on atherothrombotic risk in obese young women with polycystic ovary syndrome and controls

**DOI:** 10.1186/s12902-015-0005-6

**Published:** 2015-04-02

**Authors:** Hassan Kahal, Ahmed Aburima, Tamas Ungvari, Alan S Rigby, Anne M Coady, Rebecca V Vince, Ramzi A Ajjan, Eric S Kilpatrick, Khalid M Naseem, Stephen L Atkin

**Affiliations:** Academic Endocrinology, Diabetes and Metabolism, Hull York Medical School, Hull, UK; Centre for Cardiovascular and Metabolic Research, Hull York Medical School, Hull, UK; Department of Cardiology, Hull and East Yorkshire Hospitals NHS Trust, Hull, UK; Department of Radiology, Hull and East Yorkshire Hospitals NHS Trust, Hull, UK; Department of Sport, Exercise and Health Science, University of Hull, Hull, UK; Division of Cardiovascular and Diabetes Research, Leeds Institute for Genetics, Health and Therapeutics, University of Leeds, Multidisciplinary Cardiovascular Research Centre, Leeds, UK; Clinical Biochemistry, Hull and East Yorkshire Hospitals NHS Trust, Hull, UK; Weill Cornell Medical College Qatar, PO Box 24144, Doha, Qatar; Diabetes and Endocrinology, Diabetes Centre, York Hospital, Wigginton Road, York, YO31 8HE UK

**Keywords:** PCOS, Liraglutide, Platelet function, cIMT, Obesity

## Abstract

**Background:**

Polycystic ovary syndrome (PCOS) is associated with obesity and increased cardiovascular (CV) risk markers. In this study our aim was to assess the effects of six months treatment with liraglutide 1.8 mg od on obesity, and CV risk markers, particularly platelet function, in young obese women with PCOS compared to controls of similar age and weight.

**Methods:**

Carotid intima-media wall thickness (cIMT) was measured by B-mode ultrasonography, platelet function by flow cytometry, clot structure/lysis by turbidimetric assays and endothelial function by ELISA and post-ischaemic reactive hyperemia (RHI). Data presented as mean change (6-month – baseline) ± standard deviation.

**Results:**

Nineteen obese women with PCOS and 17 controls, of similar age and weight, were recruited; baseline atherothrombotic risk markers did not differ between the two groups. Twenty five (69.4%) participants completed the study (13 PCOS, 12 controls). At six months, weight was significantly reduced by 3.0 ± 4.2 and 3.8 ± 3.4 kg in the PCOS and control groups, respectively; with no significant difference between the two groups, P = 0.56. Similarly, HOMA-IR, triglyceride, hsCRP, urinary isoprostanes, serum endothelial adhesion markers (sP-selectin, sICAM and sVCAM), and clot lysis area were equally significantly reduced in both groups compared to baseline. Basal platelet P-selectin expression was significantly reduced at six months in controls −0.17 ± 0.26 but not PCOS −0.12 ± 0.28; between groups difference, 95% confidence interval = −0.14 – 0.26, P = 0.41. No significant changes were noted in cIMT or RHI.

**Conclusions:**

Six months treatment with liraglutide (1.8 mg od) equally affected young obese women with PCOS and controls. In both groups, liraglutide treatment was associated with 3–4% weight loss and significant reduction in atherothrombosis markers including inflammation, endothelial function and clotting. Our data support the use of liraglutide as weight loss medication in simple obesity and suggest a potential beneficial effect on platelet function and atherothrombotic risk at 6 months of treatment.

**Trial registration:**

Clinical trial reg. no. ISRCTN48560305. Date of registration 22/05/2012.

## Background

Polycystic ovary syndrome (PCOS) is the most common endocrine disorder in women of reproductive age [1]. Women with PCOS commonly present with oligomenorrhoea, hirsutism, subfertility and obesity [1]. Obesity may play a role in the aetiology of PCOS and weight loss has been found to improve many clinical features of PCOS including menses regularity and fertility [2].

Despite the lack of long term cardiovascular (CV) outcome data, PCOS has been linked to several CV risk markers including type 2 diabetes [3], increased carotid intima-media wall thickness (cIMT) [4] and impaired platelet function [5,6]. We have reported recently that obesity, rather than the PCOS phenotype, appears to have the greatest impact on atherothrombotic risk parameters [7]. Weight loss has been found to reduce many CV risk markers in PCOS including inflammation, and insulin resistance (IR) [8]. Furthermore, in a recent study in healthy obese men and women, weight loss improved platelet function, by increasing platelets’ sensitivity to the inhibitory effect of prostacyclin (PGI_2_) and nitric oxide (NO) in aggregation [9]. However, studies investigating the effect of weight loss on platelet function and cIMT in obese women with PCOS are lacking.

Lifestyle modifications and orlistat therapy are the only currently licensed treatments for obese women with PCOS not suitable for bariatric surgery. However, their efficacy is limited by the low compliance rate [10] and gastrointestinal side effects with orlistat. Recently, a glucagon like peptide-1 (GLP-1) mimetic, exenatide, has been found to improve weight and menses regularity in obese women with PCOS [11]. Liraglutide is a long acting GLP-1 analogue with 97% resemblance to the native GLP-1 and seems to be better tolerated than exenatide [12]. Similar to native GLP-1, it causes glucose dependent insulin secretion, promotes weight loss [13,14], and may subsequently improve IR. Although liraglutide is currently only licensed for the treatment of people with type 2 diabetes, it represents an attractive option for the treatment of obese women with PCOS.

The primary aim of this study was to assess whether six months treatment with liraglutide 1.8 mg once a day (od) would have an impact on body weight, platelet function and cIMT in young obese women with PCOS compared to controls of similar age and weight. Study secondary outcomes were to measure changes in anthropometric parameters, IR, lipids profile, inflammation, oxidative stress, clot structure/lysis, and endothelial function.

We hypothesised that women with PCOS and controls respond equally to treatment with liraglutide.

### Study design

Interventional study of young obese women with PCOS and controls of similar age and weight. The study was approved by the Leeds (East) Research Ethics Committee and an informed consent was given by all study participants before participation.

### Study participants

The baseline characteristics of study participants and their selection prior to treatment have been described [7]. Women with PCOS were recruited from the endocrine clinic and controls were recruited through an advertisement in the local newspaper. Subjects were invited for a screening visit if they had a body mass index (BMI) between 30–45 kg/m^2^, were between 18–45 years of age and on no medications. No one to one matching was performed. PCOS was diagnosed according to the Rotterdam criteria [15]. Participants with an alcohol intake of >14 units/week were also excluded. The metabolic syndrome was defined according to the International Diabetes Federation criteria 2006 [16]. Participants who fulfilled the study inclusion/exclusion criteria, PCOS and controls, were treated with liraglutide 0.6 mg od subcutaneous injection for 1 week, 1.2 mg od for one week and then 1.8 mg od thereafter for six months. Study participants received no dietary advice. Participants were seen at baseline, three and six months after starting the treatment.

## Methods

A detailed description of the methods used for measuring anthropometric, biochemical markers, cIMT, platelet, endothelial and clotting functions have been described [7]. A brief description is outlined below.

### Biochemical markers

Venous blood samples were collected in the morning after a minimum of a 10 h fast and samples were stored at −80°C until batch analysis. Overnight urine samples were collected and aliquots stored at −20°C until batch analysis. Biochemical markers measured included high sensitivity C-reactive protein (hsCRP), testosterone, sex hormone binding globulin (SHBG), free androgen index (FAI), lipids profile, and urinary isoprostanes (8-iso PGF_2α_). Fasting plasma glucose (FPG) and insulin were measured to calculate the homeostatic model assessment of insulin resistance (HOMA-IR): FPG (mmol/L) X fasting insulin (iu/ml))/22.5 and β-cell function (HOMA-β): 20 X fasting insulin/(FPG – 3.5) [17].

### Carotid intima-media wall thickness (cIMT)

cIMT was measured using a Toshiba Xario 15 scanner (Toshiba Medical Systems, Tokyo, Japan) equipped with 11-MHz linear imaging probe. The cIMT measurements were performed in accordance with recommendations by Mannheim consensus, 2006 [18]. All measurements were performed by the same trained operator. Reproducibility of cIMT measurements was assessed in 18 participants who underwent 2 ultrasound examinations within 4 weeks period using Bland Altman plot, mean difference = −0.002 (95% limits of agreement = −0.055, 0.059); intraclass correlation coefficient (ICC) = 0.95 (95% CI = 0.90, 0.99).

### Platelet function

Fluorescein isothiocyanate (FITC)-conjugated anti human CD42b antibody, phycoerythrin (PE)-conjugated anti human CD62P, FITC-anti IgG_1k_ and PE-anti IgG_1k_ isotope controls were obtained from BD bioscience (Oxford, UK). FITC-anti human fibrinogen was obtained from Dako (Stockport, UK). Prostacyclin I_2_ (PGI_2_) was obtained from Cayman (USA) and Adenosine 5’-diphosphate (ADP) from Sigma (Poole, UK).

Platelet function was analysed in whole blood by flow cytometry according to the methods described by Riba et al. [19]. Platelet population was identified by their forward and side-scatter characteristics and confirmed by the expression of platelet specific surface marker CD42b. P-selectin surface expression, a marker of platelet α-granule content release, and fibrinogen binding, a marker of platelet activation, were calculated from 10,000 platelet events. In brief, 5 μl of citrated blood was diluted in 50 μl of modified Tyrodes buffer (150 mM NaCl, 5 mM HEPES [N-2-hydroxyethylpiperazine-N’-2-ethanesulfonic acid], 0.55 mM NaH_2_PO_4_, 7 mM NaHCO_3_, 2.7 mM KCl, 0.5 mM MgCl_2_, 5.6 mM glucose, pH 7.4) and mixed with 2 μL of the appropriate FITC or PE labelled antibody. Samples were fixed with 500 μl of 0.2% paraformaldehyde and analysed by flow cytometry (BD FACSAria). In some cases, samples were incubated with ADP in the presence and absence of PGI_2_ before fixation.

### Endothelial function

Endothelial function was examined by measuring the post-ischaemic reactive hyperaemic index (RHI) using Endo-PAT2000 (Itamar Medical Ltd, Israel) [20]. The serum concentrations of endothelial adhesion markers sP-selectin, intercellular adhesion molecule-1 (sICAM-1), and vascular cell adhesion molecule-1 (sVCAM-1) were measured by ELISA as per manufacturer’s protocol.

### Clot formation/lysis

Clot structure analysis was measured using turbidimetric analysis as previously described [21]. The clot parameters assessed included lag phase, maximum absorbance (MA), lysis time (LT), clot formation time and lysis area (LA). Higher MA of plasma clots, longer LT and larger LA are associated increased CV risk [22].

### Power calculations and statistical analysis

The sample size for platelet function was powered using the Mann–Whitney *U* test for continuous data [23] and an ‘effect size’ between 1.12–1.25 for changes in P-selectin expression in unstimulated samples [24,25]. A sample size of 18 per group allow us to detect an effect size of 1.12 (or larger) with 80% power, 20% attrition and 5% significance (two-tailed) between cases and controls. As variations within groups are usually smaller than between groups, this study was adequately powered to detect differences’ pre/post treatment.

Continuous data were summarised by the mean ± standard deviation (SD), and categorical data by number (percentages). Data were checked for normality using Kolmogorov-Smirnov test. Differences between groups at baseline were analysed using the independent *t*-test for continuous data (or Mann–Whitney *U* test for non-normally distributed data). Frequency distributions’ were analysed using Chi-square test. Between groups’ comparisons after intervention were as follows: for each group (PCOS and controls) a difference between baseline and 6 months was calculated. The between group differences were compared using the independent *t*-test (or Mann–Whitney *U* test for non-normally distributed data).

Within groups analysis (baseline vs. six months) was performed using the dependent *t*-test for continuous data (or Wilcoxon signed-rank test for non-normally distributed data). Data were analysed using intention to treat analysis with missing values for sequential data imputed by carrying the last observation forward [26]. Statistical analysis was performed using the PASW statistics 19 package (SPSS Inc., Chicago, USA). A two tailed P value of <0.05 was considered statistically significant.

## Results

### Baseline characteristics

Fifty one women were screened, 36 recruited (19 PCOS, 17 normal controls). Fifteen women were excluded as they had; idiopathic hirsutism (five), hypothyroidism (one), BMI <30 kg/m^2^ (one), no trans-vaginal ultrasound (TV USS) scan was done (two), ovaries not visible on TV USS (one), oligomenorrhoea without PCOS (one), and repeatedly missing the initial study visit (four).

The PCOS and control groups had similar age 33.9 ± 6.7 vs. 33.5 ± 7.1 years, weight 102.1 ± 17.1 vs. 100.4 ± 15.1 kg, BMI 37.9 ± 5.0 vs. 36.5 ± 4.6 kg/m^2^, and waist 112.7 ± 12.6 vs. 112.4 ± 9.4 cm, respectively. The PCOS group had higher testosterone 1.3 ± 0.4 vs. 0.9 ± 0.3 nmol/L (P = 0.01), FAI 4.4 ± 2.0 vs. 2.6 ± 1.2 (P = 0.01), fasting insulin 22.0 ± 9.4 vs. 16.1 ± 5.6 iu/ml (P = 0.03) and HOMA-IR 5.1 ± 2.6 vs. 3.5 ± 1.3 (P = 0.03) compared to controls at baseline (Table 1). cIMT, platelet function, clot formation/lysis, and endothelial function did not significantly differ between the two groups at baseline; data recently published [7].

**Table 1:** **Anthropometric, metabolic and biochemical parameters at baseline and after treatment with liraglutide**

**Variable**	**PCOS (n = 19)**	**Controls (n = 17)**	**PCOS**	**Controls**	**Between groups difference**	
	**Baseline mean (SD)**	**Baseline mean (SD)**	**6-months-baseline**	**6-months-baseline**	**95% CI**	**P-value**
Weight (kg)	102.1 (17.1)	100.4 (15.1)	−3.0^†^	−3.8^†^	−1.9 – 3.4	0.56
BMI (kg/m^2^)	37.9 (5.0)	36.5 (4.6)	−1.0^†^	−1.4^†^	−0.6 – 1.3	0.43
Waist (cm)	112.0 (12.6)	112.3 (9.7)	−1.1	−1.4	−2.4 – 3.2	0.78
Systolic BP (mmHg)	121 ± 13.0	126 ± 12.9	0.0	−4.4	−2.9 – 11.7	0.23
Diastolic BP (mmHg)	75 (7.6)	79 (6.6)	0.3	−3.9	−2.1 – 10.4	0.19
Average HR (bpm)	67.7 (7.7)	67.3 (9.0)	3.0	4.0^†^	−5.0 – 4.0	0.77
Metabolic syndrme	10 (52.6%)	6 (35.3%)	−4.0	0.0		
Testosterone (nmol/L)	1.3 (0.4)	0.90 (0.3)*	−0.03	0.04	−0.4 – 0.2	0.63
FAI	4.4 (2.0)	2.6 (1.2)*	0.09	−0.06	−1.2 – 1.5	0.76
SHBG (nmol/L)	33.2 (14.6)	38.1 (14.4)	−2.4	2.3	−10.9 – 1.7	0.50
FPG (mmol/L)	5.1 (0.6)	4.9 (0.4)	−0.2^†^	−0.3^†^	−0.2 – 0.4	0.40
Insulin (iu/ml)	22.0 (9.4)	16.1 (5.6)*	−2.6^†^	−3.4^†^	−2.3 – 3.9	0.61
HOMA-IR	5.1 (2.6)	3.5 (1.3)*	−0.8^†^	−0.9*	−0.6 – 0.9	0.70
HOMA-β	303 (135)	255 (114)	11.2	10.7	−70.0 – 70.9	1
hsCRP (mg/L)	6.2 (8.7)	5.1 (3.4)	−1.9^†^	−1.2^†^	−4.3 – 2.9	0.21
Total chol (mmol/L)	5.0 (0.8)	5.1 (1.0)	−0.1	−0.02	−0.4 – 0.3	0.64
LDL (mmol/L)	3.2 (0.6)	3.3 (0.9)	0.06	0.08	−0.3 – 0.3	0.89
HDL (mmol/L)	1.2 (0.2)	1.3 (0.2)	−0.02	−0.04	−0.1 – 0.2	0.84
Tri (mmol/L)	1.4 (0.7)	1.4 (0.7)	−0.3^†^	−0.2^†^	−0.4 – 0.2	0.56
Chol/HDL	4.4 (0.9)	4.1 (0.8)	−0.03	0.1	−0.6 – 0.3	0.37
Isoprostane (ng/ml)	0.81 (0.53)	0.58 (0.57)	−0.5^†^	−0.4^†^	−0.5 – 0.3	0.64
Fibrinogen (mg/ml)	3.8 (1.2)	3.5 (1.0)	0.02	−0.03	−0.4 – 0.5	0.84
cIMT	0.51 (0.05)	0.48 (0.06)	0.002	0.004	−0.01 – 0.02	0.41
RHI	1.9 (0.5)	1.9 (0.5)	−0.01	0.1	−0.8 – 0.6	0.68
sP-selectin (ng/ml)	135.9 (40.3)	126.4 (25.5)	−36.4^†^	−47.1^†^	−13.7 – 35.1	0.38
sICAM-1 (ng/ml)	302.8 (53.3)	302.1 (36.9)	−53.6^†^	−78.2^†^	−16.8 – 66.0	0.24
sVCAM-1 (ng/ml)	3384 (379)	3449 (404)	−1600^†^	−1824^†^	−335 – 782	0.42

BMI, body mass index; BP, blood pressure; HR, heart rate; bpm, beat per minute; FAI, free androgen index; SHBG, sex hormone binding globulin; FPG, fasting plasma glucose; HOMA-IR, homeostatic model assessment-insulin resistance; HOMA-β, homeostatic model assessment-β cell function; hsCRP, high sensitivity C-reactive protein; LDL, low density lipoprotein; HDL, high density lipoprotein; Tri, triglycerides; Chol, cholesterol; cIMT, carotid intima-media wall thickness; RHI, reactive hyperemic index; sICAM, soluble intercellular adhesion molecule-1; sVCAM, soluble vascular cell adhesion molecule-1. Data are mean (standard deviation (SD)) or number (percentage). *Significant difference between the two groups at baseline, P < 0.05. ^†^Significant within group difference, 6-month vs. baseline, P < 0.05.

### Intervention with liraglutide

Twenty five women, 69%, completed the study (13 PCOS, and 12 controls). Eleven women dropped out because of: nausea and vomiting (four), loss of follow up (four), frequently missing study drug (one), change in personal circumstances (one), and pregnancy (one).

### Demographics

Following 6 months of treatment with liraglutide 1.8 mg od body weight was reduced by 3.0 ± 4.2 (P < 0.01) and 3.8 ± 3.4 kg (P < 0.01); BMI by 1.0 ± 1.5 (P = 0.01) and 1.4 ± 1.2 kg/m^2^ (P < 0.01); while average heart rate increased by 3 ± 7 (P = 0.051) and 4 ± 7 beats per minute (bpm) (P = 0.02) in the PCOS and control groups, respectively. There was no significant change in waist circumference, systolic and diastolic blood pressure, or in the prevalence of the metabolic syndrome (Table 1). Between groups comparisons were not significant (Table 1).

### Biochemical profile

At 6 months FPG, fasting insulin, HOMA-IR, hsCRP, triglycerides, and urinary isoprostane significantly reduced in both groups. There was no significant change in other biochemical markers including testosterone, SHBG, FAI, HOMA-β or cholesterol (Table 1). Between groups comparisons were not significant (Table 1).

### cIMT

There was no significant change in average cIMT at baseline compared to 6 months in either group. Similarly, between groups changes were not significant (Table 1).

### Platelet function

Following 6 months treatment with liraglutide there was significant inhibition of P-selectin expression in unstimulated samples in the control group, compared to baseline, without change in fibrinogen binding (Table 2). P-selectin expression and fibrinogen binding levels were unchanged in the PCOS group at 6 months compared to baseline. Similarly, platelet response to activation by ADP (0.1 - 10 μM) or to inhibition by PGI_2_ (1 - 100nM) were unchanged at 6 months in either group (Table 2). Between groups changes were not significant (Table 2).

**Table 2:** **Platelet function, activation and sensitivity to prostacyclin, and clotting function/lysis at baseline and after treatment with liraglutide**

**Variable**	**PCOS**	**Controls**	**PCOS**	**Controls**	**Between groups difference**	
	**Baseline mean (SD)**	**Baseline mean (SD)**	**6-months-baseline**	**6-months-baseline**	**95% CI**	**P-value**
Platelets P-selectin expression						
Basal activation	0.52 (0.3)	0.42 (0.2)	−0.12	−0.17†	−0.14 – 0.26	0.41
ADP 0.1 μM	3.1 (2.9)	2.8 (2.1)	−0.09	−0.16	−1.6 – 1.7	0.42
ADP 1 μM	40.2 (14.5)	44.0 (13.5)	0.2	−1.1	−4.8 – 7.3	0.74
ADP 10 μM	61.6 (13.6)	66.9 (13.1)	−0.1	2.9	−8.7 – 2.8	0.30
% inhibition by PGI_2_:						
1 nM	17.5 (14.1)	17.8 (10.9)	3.4	6.0	−9.8 – 4.6	0.80
10 nM	65.5 (11.7)	66.5 (9.6)	3.5	3.0	−7.1 – 8.1	0.67
100 nM	79.3 (6.5)	77.4 (7.1)	1.7	2.5	−5.6 – 4.0	0.74
Platelets fibrinogen binding						
Basal activation	0.97 (0.4)	0.83 (0.3)	−0.04	0.09	−0.4 – 0.13	0.31
ADP 0.1 μM	9.7 (10.0)	7.7 (7.1)	1.6	−2.6	−0.06 – 8.4	0.13
ADP 1 μM	55.9 (18.4)	55.8 (19.0)	0.6	−4.6	−3.4 – 13.8	0.30
ADP 10 μM	73.7 (14.6)	76.2 (11.7)	1.1	−4.0	−0.95 – 11.2	0.10
% inhibition by PGI_2_:						
1 nM	41.5 (23.2)	46.7 (17.6)	5.6	3.6	−13.7 – 17.6	0.90
10 nM	95.8 (2.3)	96.8 (2.1)	0.7	0.1	−0.9 – 2.5	0.28
100 nM	97.8 (1.1)	98.0 (1.4)	0.5	0.2	−0.4 – 1.7	0.21
Clot function/lysis						
Lag phase (sec)	379 (64)	380 (45)	96^†^	139^†^	−99 – 12.8	0.13
Lysis area (au)	586 (292)	538 (177)	−108^†^	−100^†^	−139 – 124	0.59
Maximum absorbance (au)	0.39 (0.1)	0.41 (0.1)	−0.01	−0.03	−0.03 – 0.06	0.49
Clot formation time (sec)	609 (190)	583 (144)	69	40	−83 – 140	0.61
Lysis time (sec)	766 (204)	743 (117)	7.1	20.9	−136 – 108	0.82

Platelet surface expression (% of positive cells) of P-selectin and fibrinogen binding were measured in unstimulated samples (basal activation) and after stimulation with ADP 0.1-10 μM for 20 minutes before fixing with 0.2% paraformaldehyde. For platelet sensitivity to PGI_2_, samples were pre incubated with PGI_2_ 1, 10, and 100 nM for 90 seconds before; stimulation with ADP 10 μM and fixing at 20 minutes for P-selectin expression; or stimulation with ADP 1 μM and fixing at 5 minutes for fibrinogen binding. Data are presented as mean (standard deviation (SD)). ADP, adenosine-5’-diphosphate; PGI_2_, prostacyclin; sec, seconds; au, arbitrary unit. Comparisons at baseline were not statistically significant (all P values were >0.05). ^†^Significant within group change, 6-month vs. baseline, P < 0.05.

### Clot function/lysis

At six months, clot lag phase significantly increased and LA reduced in both groups compared to baseline (Table 2). However, other markers did not significantly change in either group including clot LT, clot formation time, and clot MA (Table 2). There was no significant change in fibrinogen levels at 6 months compared to baseline (Table 1). Between groups changes were not significant (Table 2).

### Endothelial function

There was no significant change in RHI, at six months compared to baseline, in either group (Table 1). Serum markers of endothelial function sP-selectin, sVCAM-1 and sICAM-1 significantly reduced after treatment in both groups (Table 1). Between groups changes were not significant.

## Discussion

Our data suggest that young obese women with PCOS and controls, of similar age and weight, respond equally to treatment with liraglutide. In both groups, six months treatment with liraglutide (1.8 mg od) was associated with a small, though significant, 3 – 4% weight loss that was associated with significant reductions in IR, inflammation, oxidative stress and improvement in several CV risk markers in young obese women with and without PCOS. Our findings are important as the majority of previous reports suggest the need for moderate weight loss (≥10%) to achieve reduction in atherothrombotic risk [9,27].

The weight loss of 3 – 4% achieved in our study with liraglutide 1.8 mg od is in accord with other published data [13], and although a higher dose of liraglutide might have resulted in more pronounced weight loss [14], we have chosen the highest dose currently licensed for the treatment of people with type 2 diabetes [28]; a dose with well-known safety profile and adverse reactions. The effects of liraglutide on weight loss are thought to be mainly mediated through delayed gastric emptying and reduced appetite, rather than a change in energy expenditure [29]. While its effects on glucose metabolism are secondary to an increase in insulin secretion in a glucose-dependent manner, suppression of glucagon secretion, enhanced hepatic insulin action, and reduced β-cell apoptosis [30-32]. Similar to native GLP-1, liraglutide is widely believed to exert its actions through the GLP-1 receptor (GLP-1R) and the activation of cyclic adenosine monophosphate (cAMP) dependent pathway [28]. This is supported by the wide expression of GLP-1R including in the pancreas, stomach, and brain [32]. However, the underlying mechanisms for GLP-1 mediated weight loss remain poorly understood and may involve direct, GLP-1R mediated, and indirect, e.g. neuronal, pathways [32].

This is the first study to examine the impact of treatment with GLP-1 analogues on platelet function in obese women with or without PCOS. In this study, there was a significant inhibition in basal platelet P-selectin expression after treatment with liraglutide in the control group only. P-selectin is only expressed on the platelet surface membrane after *a* granule secretion (i.e. platelet activation) [33]. It mediates adhesion of activated platelets to neutrophils and monocytes [34]; plays a central role in platelet aggregate size and stability [35], and may play an important role in the pathogenesis of inflammation and thrombosis [35]. Subsequently, a decrease in P-selectin expression on platelet surface after treatment with liraglutide may predict a favourable CV outcome. However, the changes in platelet function noted in this study may also be related to the associated weight loss. Weight loss has been found to reduce urinary thromboxane A_2_ metabolites excretion [27] and improve platelet sensitivity to PGI_2_ in simple obesity [9]. The improvement in platelet function with weight loss was related to a reduction in abdominal obesity and associated reduction in IR, inflammation and oxidative stress levels [9,27]. Platelets have been found to express the insulin receptor [36] and while insulin is believed to reduce platelet sensitivity to aggregating agents, its function in IR states for example type 2 diabetes and obesity is thought to be impaired [37]. As PCOS, similar to type 2 diabetes, is associated with increased IR independent of obesity, it is possible that platelets from women with PCOS are more resistant to the inhibitory effects of liraglutide, and/or associated weight loss, perhaps suggesting an inherent defect in platelet function in PCOS. However, it is worth noting that between group comparisons were not significant and it is possible that the lack of significant change in basal platelet P-selectin expression in the PCOS group after treatment is related to higher than anticipated dropout during the study. While it is still not known if platelets express the GLP-1 receptor, the changes in platelet function noted in our study may still be an independent effect of liraglutide.

There was a significant reduction in clot lysis area, a complex measure of clot formation, density and lysis potential, after six months treatment with liraglutide in both groups. The majority of previous studies examining clot function and fibrinolysis in obesity reported a reduction in fibrinogen levels and reduction in plasminogen activator inhibitor-1 (PAI-1) activity after ≥ 5 – 10% weight loss [38]. Interestingly, fibrinogen levels did not significantly change in our study after treatment suggesting that the changes in clot structure parameters observed were probably related to other plasma proteins, yet to be identified; an independent effect of liraglutide; or to qualitative changes in fibrinogen induced by weight loss.

Liraglutide’s treatment did not change cIMT measurement in either group despite significant reduction in IR. This is the converse to what was reported by Orio et al. [39] who treated 30 lean women with PCOS with metformin for six months and found a significant reduction in cIMT related to an improvement in IR. In another study [40], weight loss of 3.9 kg/m^2^ over one year of dietary and lifestyle intervention in obese adolescent girls with PCOS resulted in a significant reduction in cIMT. The change in BMI in our study was smaller, 1.0 – 1.4 kg/m^2^, the follow up duration was shorter, and no diet and/or exercise interventions were included which may account for the discrepancy.

Endothelial function measured by post-ischaemic RHI was unchanged following liraglutide therapy that was discordant to the significant reduction in the endothelial serum adhesion markers sP-selectin, sVCAM, and sICAM. This is in accord with other studies showing that changes in clinical and serum markers of endothelial function in response to moderate weight loss do not always correlate. Keogh et al. reported significant improvement in sP-selectin and sICAM-1 but no change in FMD at 8 weeks despite 5-10% weight loss [41]. In another study, no change in FMD was noted despite 4 – 8% weight loss in a large cohort of obese subjects over two year period [42]. Conversely, weight loss was associated with a reduction in cIMT and improvement in FMD at 18 months and 5 years of follow up following bariatric surgery [43]. This suggests that serum markers of endothelial function are probably more sensitive to small changes in body weight, inflammation and oxidative stress that do not reflect overt functional endothelial changes.

Although treatment with liraglutide, in our study, has resulted in significant reduction in fasting plasma glucose, insulin, and insulin resistance (HOMA-IR), there was no improvement in β cell function (HOMA-β). Preclinical studies suggest that liraglutide treatment increases β cells mass [44]. Treatment with liraglutide, in a 20 week trial, increased HOMA-β by 5 – 24% in obese men and women [13]. The study included more participants (90 per group) than ours, which may explain the different results. Interestingly, there was no association between the dose of liraglutide and the change in β-cell function after treatment (median increase by 27.8% for liraglutide 1.8 mg od and 8.4% for 2.4 mg od), nor did the changes in HOMA-β correlate with HOMA-IR (which did not change during the study) [13]. It is worth noting that HOMA-β, although simple and commonly used, has many limitations including the use of a fasting parameter of β-cell function, which reflects basal rather than glucose-stimulated insulin secretion, and is also affected by alterations in insulin clearance [45-47].

Our data suggest a potential CV benefit for obese women treated with liraglutide; though how much of the changes observed during the study were related to the effects of liraglutide *per se* or to the associated weight loss remains unclear. Animal studies suggest that liraglutide may have a cardioprotective effect independent of weight loss [48].

The strengths of this study include having obese women with PCOS and controls who had similar age, weight, BMI and waist circumference; the use of well-established markers to assess atherothrombotic risk; and the first study to assess the effects of treatment with GLP-1 analogue on platelet function in obese women with and without PCOS. The main limitation to our study was the higher than anticipated dropout during the study which may have compromised the study power. We are aware that interpretation of non-significant findings is challenging. Another limitation is the absence of a placebo-treated group, with equal amount of weight loss achieved, to clarify if the changes observed were related to liraglutide *per se* or to the associated weight loss. In addition, the absence of a placebo treated group makes it difficult to completely exclude an effect of time, i.e. if the change in weight was related to regression to the mean rather than treatment with liraglutide. However, the results of this study would now allow a larger study to be powered appropriately to include a placebo.

## Conclusions

Six months treatment with liraglutide, equally affected young obese women with PCOS and controls, and was associated with mild degree of weight loss (3 – 4%) and significant reduction in IR, inflammation, and oxidative stress. In both groups, several markers of atherothrombosis also equally improved including markers of endothelial function and clot structure, but no change in cIMT was observed. While basal platelet activation was only reduced in the control group, the between groups difference was not significant. Our data support the use of liraglutide as weight loss medication in simple obesity and suggest a potential beneficial effect on platelet function and atherothrombotic risk at 6 months of treatment.
